# Comparison of two supplement oxygen methods during gastroscopy with intravenous propofol anesthesia in obese patients: study protocol for a randomized controlled trial

**DOI:** 10.1186/s13063-018-2994-8

**Published:** 2018-11-01

**Authors:** Liu-Jia-Zi Shao, Shao-Hua Liu, Fu-Kun Liu, Yi Zou, Hai-Jun Hou, Ming Tian, Fu-Shan Xue

**Affiliations:** Department of Anesthesiology, Beijing Friendship Hospital, Capital Medical University, No. 95 Yong-An Road, Xi-Cheng District, Beijing, 100050 People’s Republic of China

**Keywords:** Gastroscopy, Hypoxemia, Wei nasal jet tube

## Abstract

**Background:**

Hypoxemia is a major complication in obese patients undergoing gastrointestinal endoscopy under intravenous anesthesia or sedation due to altered airway anatomy. We design this randomized controlled trial (RCT) to compare efficacy and safety of the Wei nasal jet tube (WNJT) and nasal prongs for supplement oxygen during gastroscopy with intravenous propofol anesthesia in obese patients.

**Methods:**

The study will be a single-center, prospective RCT. A total of 308 obese patients will be recruited and randomly assigned to receive either the WNJT (group A) or nasal prongs (group B). During gastroscopy with intravenous propofol anesthesia, 5 L/min of oxygen will be delivered through the jet port of the WNJT in the group A and via the nasal prongs in the group B. The primary outcome is the incidence of hypoxemia and severe hypoxemia. The secondary outcomes are adverse events during the gastroscopy, postoperative complications, and satisfaction of the anesthetist, physician, and patient.

**Discussion:**

This RCT aims to clarify whether the WNJT can result in reduced incidences of hypoxemia and complications and provide improved satisfaction to the anesthetist, physician, and patient. Thus, it can be determined if the WNJT is a useful tool for supplement oxygen in obese patients undergoing gastroscopy with intravenous propofol anesthesia. The results will provide the evidence for anesthesiologists to make a decision regarding the choice of supplementary oxygen methods in this condition.

**Trial registration:**

Chinese Clinical Trial, ChiCTR-IOR-17013089. Registered on 23 October 2017.

**Electronic supplementary material:**

The online version of this article (10.1186/s13063-018-2994-8) contains supplementary material, which is available to authorized users.

## Background

Gastroscopy is an important and commonly used upper gastrointestinal examination. It enables physicians to visualize a variety of upper gastrointestinal lesions and is the standard tool for the diagnosis and treatment of many gastrointestinal diseases [1, 2]. However, patients are often reluctant to undergo routine gastroscopy due to its uncomfortable experience and adverse outcomes such as nausea, vomiting, anxiety, throat bleeding, etc [3]. Thus, intravenous sedation or anesthesia with short-acting intravenous anesthetics such as propofol has been recommended in the international guidelines for gastrointestinal endoscopy [4–6].

The use of intravenous sedation or anesthesia can improve patients’ comfort during gastrointestinal endoscopy, but hypoxemia is common during endoscopy with intravenous sedation or anesthesia due to respiratory depression, airway obstruction, and hemodynamic instability [7]. In particular, hypoxemia is more common in obese patients because of altered airway anatomy, such as short neck, limited neck extension, and fat deposition in the pharyngeal wall [7, 8]. As hypoxemia significantly contributes to cardiopulmonary complications, morbidity, and mortality during gastrointestinal endoscopy with intravenous sedation or anesthesia [9, 10], prevention of hypoxemia is a key step to a safe procedure. Furthermore, the international guidelines for gastrointestinal endoscopy recommend that the supplemental oxygen reduces the incidence of hypoxemia during gastrointestinal endoscopy with intravenous sedation or anesthesia [4–6].

A regular nasal cannula oxygen supply helps to reduce the incidence of hypoxemia during gastrointestinal endoscopy with intravenous sedation or anesthesia, but is inadequate for patients with increased risk factors for hypoxemia [8, 11]. Mask ventilation is difficult or even impossible during upper gastrointestinal endoscopy due to the endoscope in the mouth. Furthermore, during gastrointestinal endoscopy with intravenous sedation or anesthesia, upper airway obstruction due to soft-tissue collapse or tongue falling is a major concern [12], particularly for obese patients [7, 8]. For this reason, the placement of a nasopharyngeal airway may be a good solution as it can be conveniently inserted and ensures an open airway [13]. A prospective randomized study by Xiao et al. [14] evaluated the efficacy and safety of the nasopharyngeal airway relative to the nasal cannula in obese patients undergoing gastroscopy with intravenous anesthesia and showed that the amount of reduction in pulse oxygen saturation (SpO_2_) from baseline during gastroscopy was significantly less in the nasopharyngeal airway group (6.03%) than in the nasal oxygen tube group (10.46%).

**Fig. 1:**
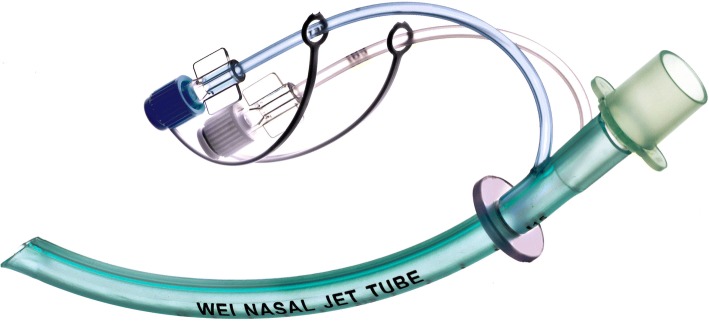
Wei nasal jet tube

The Wei nasal jet tube (WNJT; Well Lead Medical Co. Ltd., Guangzhou, China; Fig. 1) is a new special nasopharyngeal airway. Compared with the convenient nasopharyngeal airway, the WNJT can be connected to an anesthesia machine directly and pure oxygen can be delivered through its jet port. Furthermore, the WNJT has a small channel built inside the tube wall for monitoring the end-tidal partial concentration of carbon dioxide (P_ET_CO_2_), which can be used as a sign to identify the existence of a smooth flow in the airway and the occurrence of respiratory depression during gastroscopy with intravenous anesthesia or sedation [15, 16]. Currently, there are two types of commercial WNJTs available for adult patients, with the inner diameters of 5.0 mm and 7.0 mm, the outer diameters of 7.3 mm and 10.0 mm, and the lengths of 145 mm and 155 mm.

In the available literature, one multicenter, randomized controlled trial (RCT) has assessed the influences of supplement oxygen with the WNJT on respiration and ventilation during upper gastrointestinal endoscopy with propofol sedation in patients with a normal body mass index. This study shows that supplement oxygen with the WNJT compared with nasal cannula oxygen can significantly decrease the use of jaw-thrust maneuver but does not affect the incidences of total adverse events, subclinical respiratory depression, hypoxia, severe hypoxia, and mask ventilation [15]. Furthermore, it has been shown that the supraglottic jet ventilation by the WNJT during upper gastrointestinal endoscopy for patients who are sedated with propofol reduces the incidence of hypoxia in normal patients [15] and a morbidly obese patient [17]. However, there has been no study having determined the influences of supplement oxygen with the WNJT on respiration and ventilation during upper gastrointestinal endoscopy with propofol sedation or anesthesia in obese patients. In addition, placement of the WNJT is an invasive procedure and may result in a potential risk of nasal injury and bleeding [14]. Considering that there are limited data on the use of the WNJT during upper gastrointestinal endoscopy with intravenous sedation or anesthesia in obese patients and no study has assessed whether the WNJT performs better than the convenient nasal prongs for supplement oxygen in this condition, this RCT is designed to compare the efficacy and safety of the WNJT versus nasal prongs for supplementary oxygen during gastroscopy with intravenous propofol anesthesia in obese patients.

## Methods/design

**Fig. 2:**
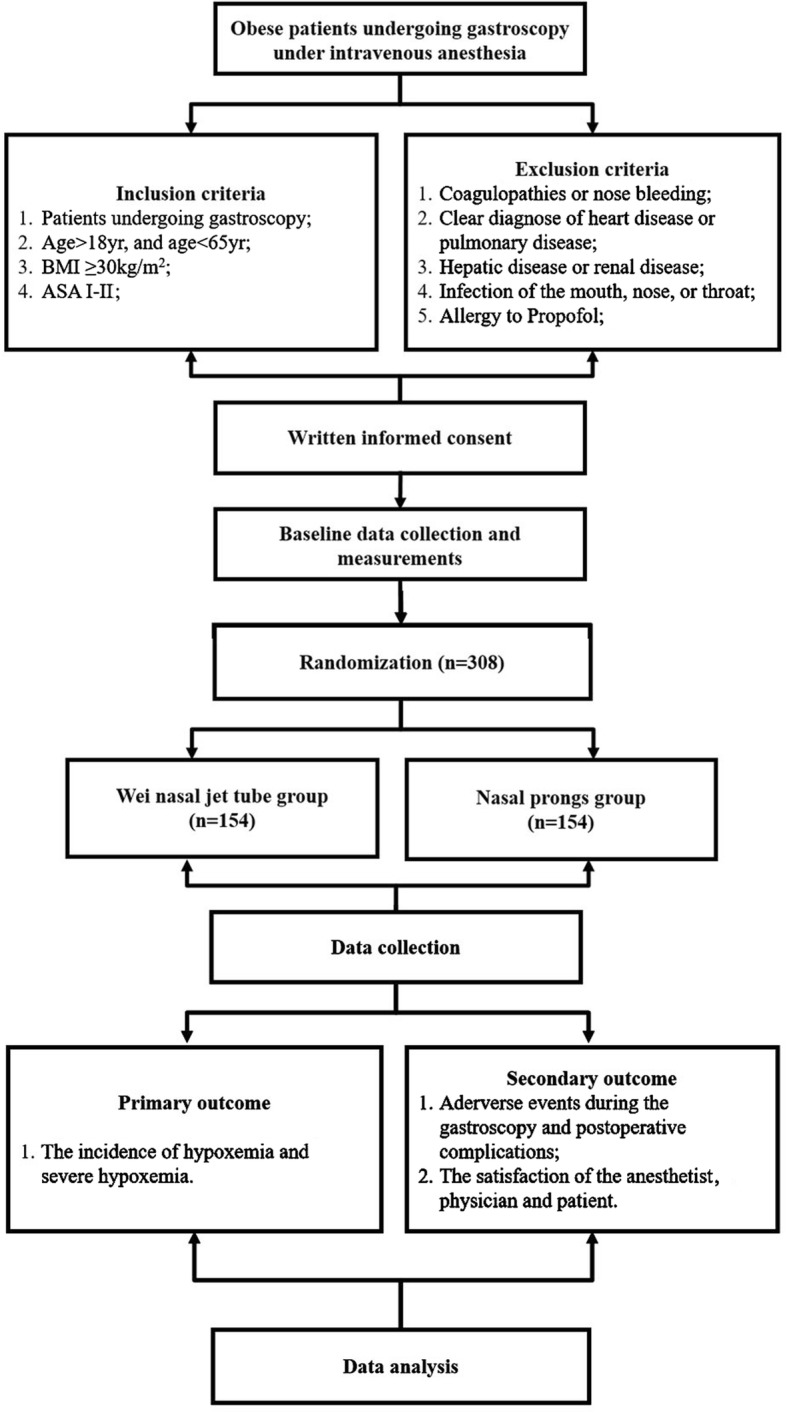
*Flow chart* of present study

**Fig. 3:**
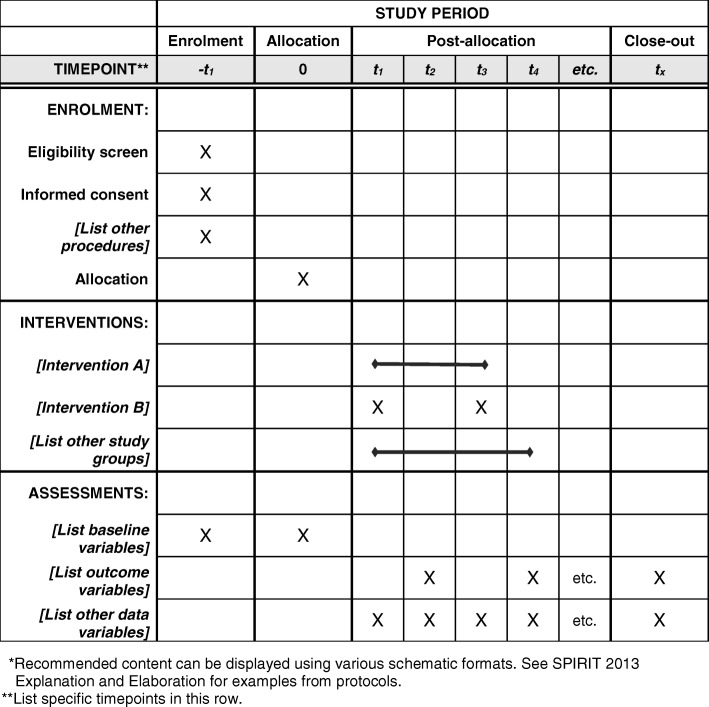
The SPIRIT figure

The flow chart of this study is shown in the Fig. 2.The SPIRIT Checklist and Figure are presented as Additional file 1 and Fig. 3, respectively.

### Trial design

This study is a single-blinded, prospective RCT. The protocol has been approved by the Ethics Committee of Beijing Friendship Hospital, Capital Medical University, Beijing, China (Ethics Committee number: 2017-P2–009-02) and is registered with Chinese Clinical Trial Registry (http://www.chictr.org.cn/; registration no. ChiCTR-IOR-17013089). The patients will be recruited from the Gastroenterology Clinic of Beijing Friendship Hospital. All patients who participate in the study must provide their written informed consent.

### Setting

The Gastroenteroscopy Center of Beijing Friendship Hospital, Capital Medical University, Beijing, China.

### Participants

The inclusion criteria are male or female patients aged 18–65 years, with the American Society of Anesthesiologists (ASA) physical status classifications I–II and BMI ≥ 30 kg/m^2^. Exclusion criteria are: coagulopathy or nose bleeding; severe cardiac, pulmonary, hepatic, or renal diseases; infection of the mouth, nose, or throat; allergy to propofol, eggs, soybean, or albumin, and others.

### Randomization and blinding

According to a random number table generated by a computer, patients are randomly assigned to receive either the WNJT (group A) or the nasal prongs (group B). An online random list generation will be utilized to perform blocked randomization in a 1:1 ratio. Outcome assessment and statistical analysis will be performed by independent researchers who are from the Clinical Research Institute of Beijing Friendship Hospital and are blind to the group assignment.

### Interventions

All patients are fasted for 8 h before the gastroscopy. After patients enter into the examination room, a topical anesthesia of oral cavity and pharynx is performed by gargling 2% lidocaine gel (10 g:0.2 g; Jumcan Pharmaceutical Group, China); two sprays of ephedrine to the nasal mucosa of each nostril are applied in all patients as to our practice. Heart rate (HR), blood pressure (BP), and SpO_2_ are continuously monitored. After establishing an intravenous access, patient is placed at the lateral position as is the routine practice of our hospital for gastrointestinal endoscopy. Preoxygenation is then performed until an end-tidal oxygen concentration of 88–90% is reached. To facilitate preoxygenation, the patient is intermittently asked to take eight deep breaths in 60 s with 100% oxygen [18]. The end-tidal concentrations of oxygen and carbon dioxide during preoxygenation are continuously monitored by a gas analyzer of anesthesia machine.

After adequate preoxygenation, anesthesia is induced with intravenous injection of propofol 2 mg/kg (10 mg/1 mL; Diprivan, Astrazeneca, UK). The depth of anesthesia is assessed by an anesthesiologist blinded to the group assignment. During the gastroscopy, 20–30 mg of propofol are supplemented as necessary to maintain an Observer’s Assessment of Alertness/Sedation score of 2 or 3 [19]. For patients receiving the WNJT, an appropriate size (ID 5.0 or 7.0 mm) device is selected by examining the outer diameter of the device and the patient’s nostrils. The device that is large enough but can be easily inserted through the nostril is selected. The WNJT is lubricated with 2% lidocaine gel (Jumcan Pharmaceutical Group, China) before insertion. The distance between the tip of the nose and earlobe on one side is measured using the scale on the exterior wall of the WNJT. After adequate anesthesia is achieved, the WNJT is inserted to the measured depth in the group A and the nasal prongs are placed in the group B. The position of the WNJT is re-examined by gastroscopy and adjusted for appropriate if necessary. The ideal position is designed as the distal tip of the WNJT is within 1 cm of the epiglottis tip. If insertion of WNJT is difficult via the selected nasal passage, the other side can be tried. If it is still unsuccessful after three attempts, insertion of the WNJT is regarded as a failure. During the gastroscopy, 5 L/min of oxygen is delivered directly through the jet port of the WNJT without using a jet ventilator in the group A and via the nasal prongs in the group B, respectively. Both the WNJT and nasal prongs are removed before the patients recover consciousness. Consequently, the patients are also blinded to the grouping assignment.

Throughout the gastroscopy, if hypoxemia (SpO_2_ < 90%) occurs, the taken measures include [4–6]: (1) audio or painful stimulation; (2) no additional drugs; (3) increasing the volume of oxygen from 5 to 8 L/min; (4) airway opening with the jaw-thrust maneuver; (5) removing the gastroscope tube and performing facemask ventilation; and (6) tracheal intubation for mechanical ventilation if necessary.

During the study, if nasal bleeding occurs in the group A, compression hemostasis is first performed; if it does not work, other medical or surgical measures should be considered.

### Data collection

#### Patients’ information

Demographic data of patients—including age, gender, body mass index, history of hypertension, diabetes, and coronary artery disease—are collected from the medical records. Airway conditions, ASA physical status classifications, BP, HR, and SpO_2_ before anesthesia are assessed and recorded by a research assistant.

#### Data about the procedure

The BP, HR, and SpO_2_ during the endoscopy are recorded every 2 min. Total propofol dose, recovery time, hypoxemia, severe hypoxemia, times required for use of assistant airway maneuvers (jaw-thrust, mask ventilation, and tracheal intubation), adverse events during the endoscopy procedure (body movement, vomiting, regurgitation and aspiration, bronchospasm, cough, bradycardia, tachycardia, hypertension, hypotension), nasal bleeding, and recovery delay are also recorded.

In this study, hypoxemia is defined as SpO_2_ of 75–89% for < 60 s, and severe hypoxemia is defined as SpO_2_ < 75% at any time or SpO_2_ of < 90% for > 60 s [20, 21]. Hypotension is defined as a decrease in mean arterial pressure (MAP) > 20% or non-invasive blood pressure of not more than 90/60 mmHg; hypertension is defined as an increase in MAP > 20% or non-invasive blood pressure of at least 140/90 mmHg; bradycardia is diagnosed if HR is < 50 beats/min; tachycardia is diagnosed when HR stays above 100 beats/min. If necessary, ephedrine or phenylephrine, urapidil, atropine, and esmolol were intravenously injected to treat hypotension, hypotension, bradycardia and tachycardia, respectively [21–23].

Nasal bleeding is assessed using a subjective scale: 0, no bleeding; 1, minimal bleeding not requiring suctioning; 2, moderate requiring suctioning but not hampering visualization, and 3, severe requiring suctioning and hampering visualization [24].

#### Data after the procedure

Satisfaction of the anesthetist, physician, and patient is assessed using a 10-point visual analog scale at the time of patient’s consciousness recovery and 30 min after consciousness recovery, respectively, and classed as follows: poor, 1–4; fair, 5–7; and good, 8–10 [25]. Adverse events such as sore throat, nasal bleeding, nausea, and vomiting are also collected at the time when the patient recovers consciousness and 30 min after the recovery of consciousness.

### Study outcomes

#### Primary outcome

The primary outcome is the incidence of hypoxemia and severe hypoxemia [20].

#### Secondary outcomes

The secondary outcomes are the adverse events during the gastroscopy, nasal bleeding or injury, complications, and satisfaction of the anesthetist, physician, and patient.

### Sample size estimation and statistical analysis

The sample size is calculated with the Pass software (version 11.0, NCSS, LLC, Kaysville, UT, USA). The two independent proportions procedure is used. With an α = 0.05 and a power of 80%, we estimated that 140 patients per group would be required for our study. If the dropout rate is set at about 10%, a total of 308 patients (154 in each group) would be required. The incidence of hypoxemia during gastroscopy in obese patients with intravenous propofol anesthesia has been reported to be 22% [20]. Thus, a proportion of 22% of the patients in the group B is expected to develop hypoxemia. P1 and P2 are calculated from the assumption that the WNJT would achieve a reduction from 22% to 11% for the incidence of hypoxemia.

Statistical analysis of data will be performed using the SPSS (Version 23.0, SPSS Inc., Chicago, IL, USA) by a blinded statistician. Continuous variables will be presented as mean ± standard deviation (normally distributed data) or medians and ranges (non-normally distributed data). Numerical data will be compared using a Student’s t-test (normally distributed data) or Mann–Whitney rank sum test (non-normally distributed data). Qualitative data will be expressed as n (%) and compared using a Chi-squared test. A *P* value < 0.05 is considered statistically significant.

### Handling of data

Study data are collected and managed by one statistician and checked by another statistician from the Clinical Research Institute of Beijing Friendship Hospital to promote data quality. An interim analysis will be performed by a data and safety monitoring board when half of the patients are enrolled. By this analysis, the data and safety monitoring board can advise on adjusting the study conduct, design, and others. Full access to the final trial dataset will be granted to the selected investigators only (YZ and HJH).

### Study dropouts

Investigators have the right to terminate participation of any individuals at any time if the investigator deems it in the participant’s best interest. Furthermore, the individual has the right to withdraw the consent to participate in the study at any time for any reason without any consequences for further medical treatment. The study discontinuation will be documented.

## Discussion

Hypoxemia caused by transient respiratory inhibition and airway obstruction is common during gastroscopy with intravenous anesthesia or sedation, especially in obese patients due to altered anatomy of the airway, reduced functional residual volume of the lungs, and decreased compliance of the chest wall [12]. Although respiratory depression may be transient and spontaneously recover with or without intervention, it can result in a risk of interrupting or stopping the gastroscopic procedure and even lethal hypoxemia requiring emergent management. The nasopharyngeal airway can prevent airway obstruction caused by the falling tongue and reduce air flow resistance, thus facilitating spontaneous breathing and decreasing the occurrence of hypoxemia during gastroscopy with intravenous anesthesia or sedation [26].

This RCT is designed to test the hypothesis that the WNJT, a new kind of nasopharyngeal airway, can reduce the incidence of hypoxemia and related adverse events. We believe that the findings of this study will have significant clinical implications; even negative results are obtained. This would mean that more studies are needed to explore a safe and effective method to prevent hypoxemia during gastroscopy with intravenous anesthesia or sedation in obese patients. This is one of the main strengths of present study.

There are some issues in our study design that need to be noted. First, this is a single-blinded protocol, as the investigator knows whether nasal prongs or the WNJT are used. This may bias the findings of this study. Second, as the nasal soft tissue is easily damaged [27], the WNJT may lead to discomfort and even nasal bleeding or injury, as reported in a previous study [15]. Furthermore, the nasal bleeding or injury may only occur in the group A. Although the nasal bleeding and injury associated with nasopharyngeal airway are often slight, care should be taken to avoid this; essential management is required if they occur, especially for patients with nasal stenosis [28].

In summary, this single-center, prospective RCT will compare the efficacy and safety of using the WNJT and nasal prongs for supplemental oxygen during gastroscopy with intravenous propofol anesthesia in obese patients. The result of the trial will provide the evidence for anesthesiologists to make a decision regarding the choice of supplemental oxygen methods in this condition.

## Trial status

The trial is currently recruiting participants. To date (10 May 2018), 20 participants have been recruited. A report releasing study results will be submitted for publication in an appropriate journal, approximately 10 months after finishing data collection and analysis.

## Additional file

Additional file 1: The SPIRIT checklist. (DOC 132 kb)

## Abbreviations

BP: Blood pressure
HR: Heart rate
MAP: Mean arterial pressure
SpO_2_: Pulse oxygen saturation
WNJT: Wei nasal jet tube
